# What Is the Consensus from Multiple Conclusions of Future Crop Yield Changes Affected by Climate Change in China?

**DOI:** 10.3390/ijerph17249241

**Published:** 2020-12-10

**Authors:** Chengfang Huang, Ning Li, Zhengtao Zhang, Yuan Liu, Xi Chen, Fang Wang, Qiong Chen

**Affiliations:** 1 The Key Laboratory of Environmental Change and Natural Disaster, Ministry of Education, Beijing Normal University, Beijing 100875, China; huang_cf@mail.bnu.edu.cn (C.H.); ningli@bnu.edu.cn (N.L.); 201831051088@mail.bnu.edu.cn (Y.L.); chen_xi0512@mail.bnu.edu.cn (X.C.); wangfang@mail.bnu.edu.cn (F.W.); 2 Academy of Disaster Reduction and Emergency Management, Ministry of Emergency Management & Ministry of Education, Faculty of Geographical Science, Beijing Normal University, Beijing 100875, China; 3 Academy of Plateau Science and Sustainability, Xining 810008, China; qhchenqiong@163.com

**Keywords:** consensus, climate change, yields change, China

## Abstract

Many studies have shown that climate change has a significant impact on crop yield in China, while results have varied due to uncertain factors. This study has drawn a highly consistent consensus from the scientific evidence based on numerous existing studies. By a highly rational systematic review methodology, we obtained 737 result samples with the theme of climate change affecting China’s crop yields. Then, we used likelihood scale and trend analysis methods to quantify the consensus level and uncertainty interval of these samples. The results showed that: (i) The crop yield decrease in the second half of the 21st century will be greater than 5% of that in the first half. (ii) The crop most affected by climate change will be maize, with the decreased value exceeding −25% at the end of this century, followed by rice and wheat exceeding −10% and −5%. (iii) The positive impact of CO_2_ on crop yield will change by nearly 10%. Our conclusions clarify the consensus of the impact of future climate change on China’s crop yield, and this study helps exclude the differences and examine the policies and actions that China has taken and should take in response to climate change.

## 1. Introduction

Climate change has many elements and affects biological and human systems in different ways [1]. The agricultural sector is highly vulnerable to climate change [2]. Climate change can reduce agricultural yields, resulting in food insecurity, which directly affects people’s livelihoods [3]. The second sustainable development goal (SDG) of the United Nations is to end hunger, achieve food security, improve nutrition and promote sustainable agriculture. Climate change could potentially interrupt progress toward a world without hunger [4]. Identifying the particular crops that have been most affected by climate change would help with the efforts to measure and analyze ongoing efforts to adapt the change [5]. Therefore, estimating future crop yields can provide important theoretical and data support for achieving this SDG.

To study the effects of climate change on crop yields, one of the main methods is to quantify the correlation between historical climatic factors and crop yields through correlation analysis methods and then use this information to estimate future crop yields [6,7,8]. The other approach is to use the climatic factors of future climate scenarios to drive crop models and to adjust the accuracy of the model parameters in order to assess the impact of climate change on crop yields [9,10]. However, these methods are limited by assumptions about energy, population, and economics, which constrain the prediction of the climate factors that are used to predict global warming; furthermore, different general climate models (GCMs) predict differences in regional climate change [11]. In addition, there is still some room to further develop our understanding of the input data quality of crop models, the crop growth characteristics, and the coupling mechanism between management and the environment [12,13]. Therefore, due to the differences in the spatial distribution, crop model, data acquisition and other factors, results from different studies on the impact of how crop yields change under climate change are highly uncertain, and as a result, there is not a unified and reliable conclusion of crop yield change under future climate change from the existing numerous studies.

To summarize the credible conclusion of these findings from different articles, based on a qualitative expression of the consistency of basic evidence, the IPCC (The Intergovernmental Panel on Climate Change) Fifth Assessment Report (AR5) adopts methods such as statistical models to evaluate confidence, which indicates the degree of consensus and recognizes any uncertainty in each conclusion [14]. Based on many crop yield results and given the stage of climate change, AR5 notes that high-confidence research shows that negative impacts of climate change on crop yields have been more common than positive impacts.

An analysis of existing studies found that, in China, these uncertainties will be magnified by many factors, such as crop types [15,16], planting areas [17,18], climate scenarios [11,19], crop models [15,20], and CO_2_ fertilization effects [21,22]. There are many studies on the impact of climate change on Chinese crop yields, but each study has a different baseline, quantitative expression of conclusions, climatic scenario, time period, and spatial scale. For example, Liu et al. [7] predicted that maize yields in China would decrease by approximately 0.67% from 2040 to 2060, while Xiong et al. [23] estimated that the changes would be approximately −8.6% in 2050. The difference between the two yield changes is approximately 8%. China’s third National Assessment Report on Climate Change noted that the average temperature in China increased (0.21–0.25 °C/10 a) and was significantly higher than the global rate of change. As a result, future agricultural security in China is more sensitive and vulnerable to climate change than that in other areas. Assessing the consensus of conclusions is a useful way to summarize the range of projected outcomes and to combine and compare the results from numerous studies [24]. Therefore, it is necessary to unify the understanding of the impact of climate change on the yield changes in China based on the scientific evidence of numerous studies and to conclude the agreed conclusions.

A highly rational systematic review methodology to extract scientific evidence from numerous articles were used in this paper, to summarize the impact of climate change on China’s major crop yields (maize, rice and wheat). By searching, screening and reviewing many published articles, and then extracting and summarizing their research results, finally, we analyzed the conclusion consistency of these result samples, so as to reach a unified and reliable conclusion, that is the high-consensus conclusions of the future yield change under the same conditions. The results of this study intend to provide a comprehensive summary of the impact of climate change on crop yields in China and to determine the consensus of the conclusions of different articles. The high-consensus conclusions are conducive to providing scientific theoretical support for agricultural responses to climate change and to promoting the sustainable development of future crop yields; furthermore, this approach provides more unified and credible data support, based on crop yields, for investigating the impacts of climate change on the future population and economic situation.

## 2. Materials and Methods

### 2.1. Data Collection

We used a highly rational systematic review methodology to extract scientific evidence from numerous articles. First of all, we identified the keywords (climate change/climatic change/climate changing/crop/corn/maize/rice/wheat/yield/China), research themes (Chinese crop yield under climate change) and literature databases (Web of Science (WOS, Search websites: apps.webofknowledge.com), the China National Knowledge Infrastructure (CNKI, Search websites: www.cnki.net)) to search for articles published after 2007, and we obtained 1245 literature sources on 18 January 2019.

For the systematic review methodology, we established some “inclusion criteria (IC)” for constraining these obtained articles. The main contents of the IC are as follows: (i) Object: three main crops (maize, rice, and/or wheat) in China; (ii) Approach: crop process models or statistical analysis methods; (iii) Indicator: projected climate change from 2020 to 2099 with temperature, precipitation and CO_2_ fertilization as the main climatic variables, production technology and planting mode to improve crop yield are not considered; (iv) Conclusion: the yield change stated in the article’s results must be related to a certain year or period in the future scenario, rather than on temperature and precipitation.

**Figure 1:**
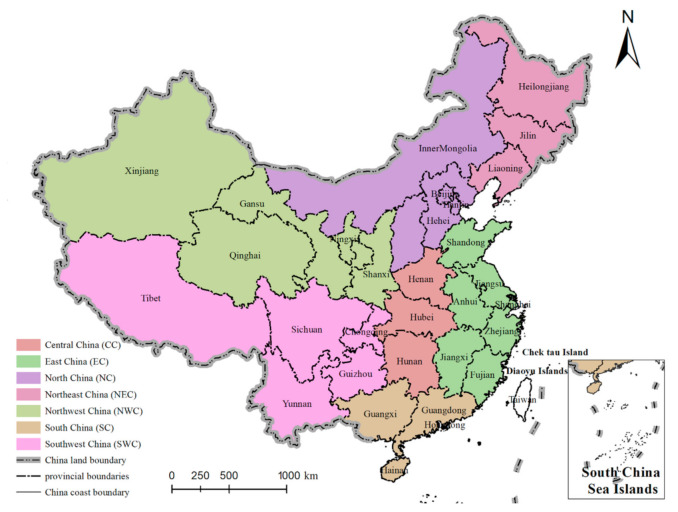
The 7 regions of China (except Taiwan, Hong Kong and Macau). (The different colors in the figure represent different regions. In each region, there are its provinces, and the label on the map is the name of the province.)

Following the above IC, our initial filtered approach was based on titles and then on abstracts, and then we investigated the full text in the rest of the articles. We selected 21 articles in the end, and the 21 selected articles (SAs) are shown in the supplementary materials (Table S1). By reading the content and analyzing the research results of the 21 SAs, we obtained 737 samples of conclusions which corresponding to yield change by the three crop types, different regions (the distribution of the regions are shown in Figure 1) and inconsistent predicted time periods (a detailed description of the process is provided in the supplementary materials File S1). Table 1 shows the statistics of the conclusion sample size.

### 2.2. Standard Setting of Consensus

The IPCC’s confidence level uses five qualifiers: very low, low, medium, high, and very high. This paper applied Mastrandrea’s [14] likelihood scale (see Table 2) to quantitatively measure the level of consensus. We used the probability scale to quantify the uncertainty, and the quantitative probability values in this paper were based on the consensus of different articles regarding yield changes in a certain year. For example, if all the conclusions in the SAs indicated that 60% of the data considered the 2050s wheat yield changes to be between 0% and −5%, we could believe that the wheat yield would be likely to fall by 0–5% in the 2050s (60%; referring to Table 2 medium consensus level, MC).

## 3. Results

### 3.1. Difference and Consensus Analysis

**Figure 2:**
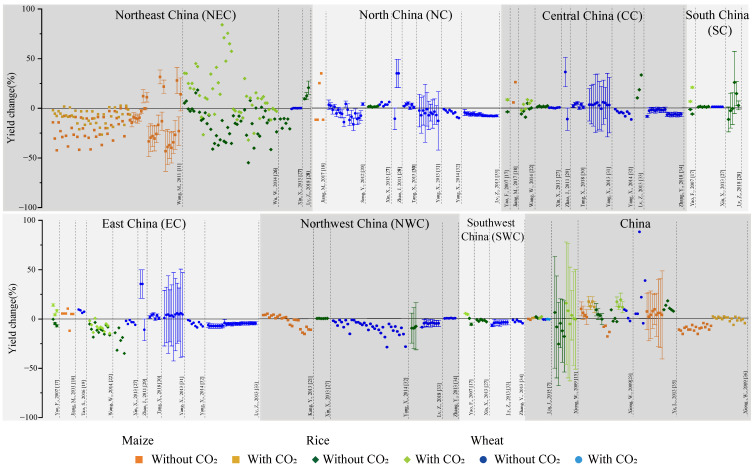
Summary of sample values of conclusion from selected articles (SAs) [7,11,15,17,18,19,20,21,22,23,26,27,28,29,30,31,32,33,34,35,36] on yield changes in China and 7 regions. (This figure shows the conclusions from all SAs. Different symbols and colors represent different crops, and the shade of the color indicates whether the CO_2_ factor was considered, as stated in the legend in the figure. The contents of the horizontal coordinate annotation are the researcher (sorted by the author’s initials) and the publication date of the article corresponding to the data in the figure. The ordinate represents the percent change in yield. Grayscale blocks represent individual study areas, corresponding to Figure 2).

By analyzing and summarizing the data sets, we compiled the graphs shown in Figure 2. Due to differences in topography, climate, and environment, there is large spatial heterogeneity in the distribution of crops in China [25]. The 7 regions differ based on the amount of sample and the type of crops.

In terms of the number of samples, the southwest (SWC) and south China (SC) are relatively small, while the northeast China (NEC) is the largest, and there are also many samples in the whole country. In terms of the number of the SAs, north China (NC), east China (EC) and central China (CC) have the most research articles, with 8,10 and 10 articles respectively. From the perspective of crop type, the sample size of wheat results is the largest, and the study regions for it are widely distributed. Consistent with the distribution of maize planting in China [37], there are few studies on maize yield change in SC, northwest China (NWC) and SWC. In addition, it can be seen from Figure 2 that there is not a one-to-one correspondence between the number of articles and the result sample size. For example, in NEC, although there are only 4 SAs, the result sample size is the largest, which is closely related to the research objectives of these research-based articles. Wang et al. [11] modified a site-based biophysical model to a spatial grid-based application and a combination of 20 general circulation models and 6 scenarios to explore the future corn yield in Jilin, and they obtained 84 data units for the 2020s, 2050s and 2070s

According to Figure 2, the future yield variation trend of different crops in different regions of China can be simply analyzed. From the distribution of sample values, it can be seen that there are obvious fluctuations in future yield changes, and most of the sample values are below the horizontal line of 0 value (Yield change = 0), in other words, there is an obviously decrease trend of the yield changes. CO_2_ factor has obvious positive effect to future yield change, performance in the same article for the same crop yield in a certain period of changing values under the same condition of future climate change; for example, in Figure 2, the results of the first article on future maize yield changes were all higher with considering the effects of CO_2_ than those without considering CO_2_ factors, as with the second article for rice and other SAs for different crops. From the perspective of different crops, there was a high degree of consensus on the future yield change of wheat, and most of the sample values of it show a decrease trend, but the range of yield decrease is small, although some of the sample values show an increase trend, and their uncertainty range are also below the horizontal line of 0 value. For maize and rice, the regional characteristics of yield change are obvious, although limited by the number of articles, the overall trend was mainly decrease. In addition, it can be found that most of the samples give a range of uncertainty, perhaps due to the limitations of research methods or data in different SAs, and there are differences in the estimated changes of the same crop yield in the same region; for example, Yang et al. [31] and Song et al. [20] both studied the wheat yield changes in NC, and their conclusions were −5.6% and −6.5% (mean values for sample values of conclusion in their studies), respectively; however, the uncertainty range of the former was ±17%, while that of the latter was ±6.5%.

### 3.2. Consensus Analysis of the Temporal Trend

#### 3.2.1. Tendency of Yield Changes

**Figure 3:**
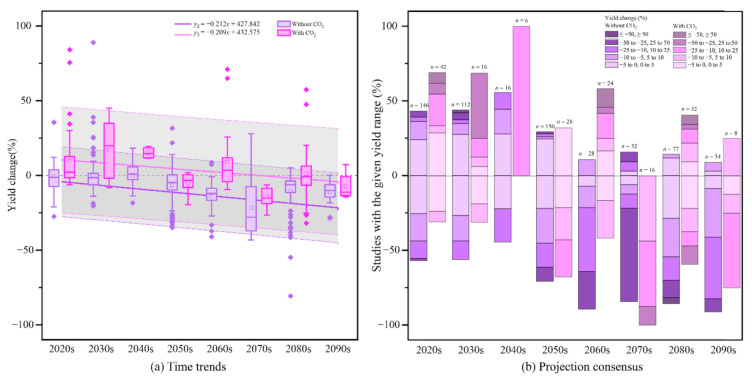
Temporal trend and results of the consensus of crop yield changes in China under climate change. ((**a**) Box plot showing the temporal trend of the yield change results; the middle of the box plot represents the median; the square represents the mean; the colors indicate the results of whether CO_2_ factors are considered (pink is with, and purple is without). The shaded portion of the trend line in the figure indicates the uncertainty interval, which is ±23% without CO_2_ and ±35% with CO_2_. (**b**) Bar graph showing the range of consensus levels for the results of crop yield changes, with the n value indicating the number of results for the estimated crop yield for that era).

The main trend of the crop yield change in China showed a significant decrease (linear regression line in Figure 3a). Without considering the impact of CO_2_, the crop yields in China decreased by 4.09% (the average value of the yield change results, the same below) from that in the 2020s, and the rate of yield change to the 2050s exceeded −10% (yield change was −11.59% in the 2050s) and −20% in the 2090s (−22.84% in the 2090s).

However, when considering the impact of CO_2_, before the 2070s the crop yield change will be mainly positive, but the amplitude of change gradually decreases, and at the same time, the yield decreases by 2% in the 2080s. In addition, the regression trend analysis of the yield changes over time showed that the trend line with CO_2_ was higher than that without CO_2_, and the rate of yield change with CO_2_ was greater, as shown, with the rate of yield change approximately −2.5% per 10 years without CO_2_, while it was approximately −2% per 10 years with CO_2_. Meanwhile, the uncertainty of the yield change with CO_2_ was larger, the figure shows that the uncertainty range of the yield change was ± 23% without CO_2_ but ± 35% with CO_2_.

Whether CO_2_ factors had the opposite effect on extreme values was considered, the 2070s can be thought of as a period of demarcation. Before the 2070s, the extreme values of yield change without CO_2_ will have positive and negative directions, and over time, the positive value decreases, and the negative value increases; however, after the 2070s, the extreme value is only negative. After considering the influence of the CO_2_ factor, the extreme value is opposite to the results without CO_2_, i.e., the yield change is positive before the 2070s, and the positive value is decreasing, although the directions become positive and negative after the 2070s.

#### 3.2.2. Consensus Trend of Yield Change

Although future yield changes are limited by many uncertainties, numerous research conclusions have been temporally dependent (Figure 3b). There is a majority consensus in that the yield changes will be negative from the 2020s onwards. The magnitude of the yield impact generally increases with time: more than 60% of projections indicate that the yield changes in the 2030s will be −5–5%, and the projections of the decreasing yield gradually increase after the 2050s; finally, 65% of the projections indicate that the yield decreases in the 2070s are greater than 25% (MC). Hence, for China, the scientific experiments and research on improving crop planting technology and cultivating new crop cultivars needs to be carried out in a targeted way, and it is necessary to prevent an even greater decline in yield after 2050 as soon as possible.

The impact of CO_2_ factors on yield changes is reflected by two aspects: the projections of yield changes and the level of consensus on the projections. Without considering the CO_2_ factor, most of the projections show yield decreases of approximately 6.6% (MC) in the 2020–2040s and yield decreases of approximately 11.6% (LC), 14.1% (MC), 16.6% (MC), 19.1% (LC), 22.8% (MC) in the five decades of the second half of the century. On the other hand, under the impact of CO_2_, the yield changes are approximately +8.46% (HC) in the 2020–2040s; the yield change begins to decline until 2080, i.e., by approximately −1.99% (MC) in the 2080s; and in the 2090s, the yield decreases by 5.19% (MC).

### 3.3. Consensus Analysis of Temporal Trends with Different Crops

#### 3.3.1. Tendency of Yield Changes

**Figure 4:**
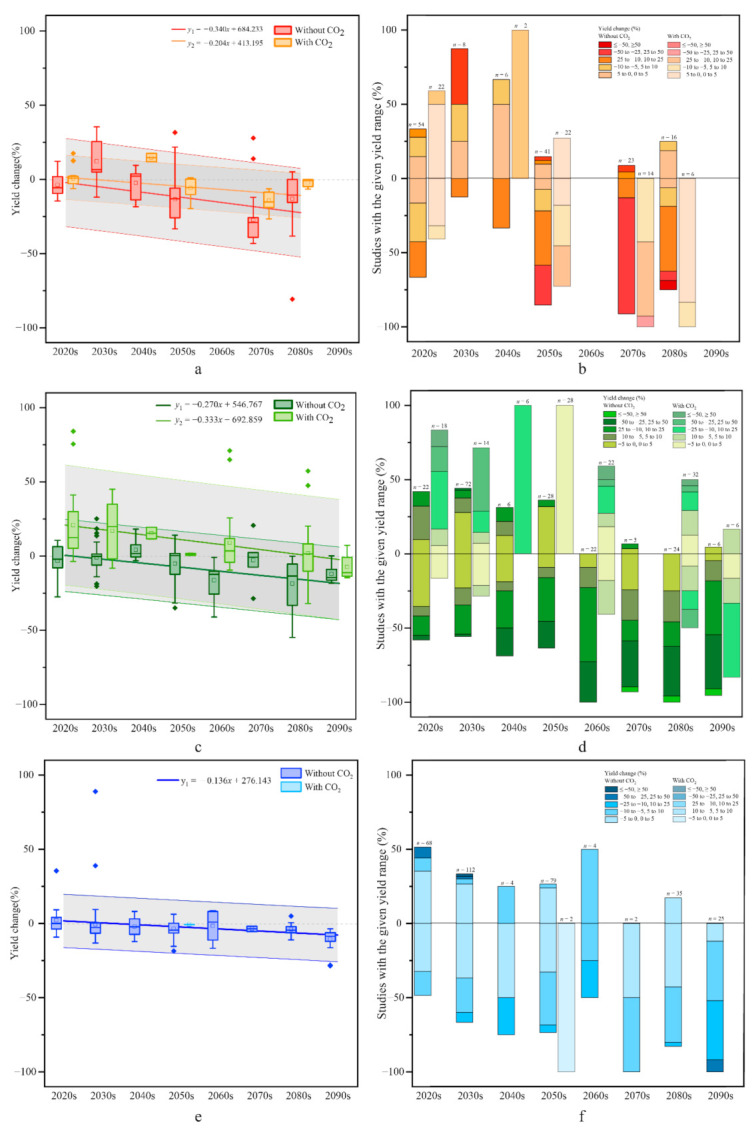
Temporal trend and consensus of maize (**a**,**b**), rice (**c**,**d**) and wheat (**e**,**f**) yield changes in China under climate change. (The meaning of each component in the figure is the same as that in Figure 4, but different colors are used to represent different crops. In addition, the shaded portion of the trend lines in Figure 4a/c/e indicates the uncertainty interval, which is ±16%, ±24% and ±18% without CO_2_ for maize, rice and wheat and ±35% and ±40% with CO_2_ for maize and rice, respectively).

From the trend analysis, there was a phenomenon in which the yield changes of maize (Figure 4a), rice (Figure 4c), and wheat (Figure 4e) in China all decreased over time. Moreover, maize and rice were significantly affected by CO_2_ factors, and there is a lack of research on wheat yield changes with CO_2_.

The maize yield changes varied greatly with decade. However, the CO_2_ factor had a positive impact on the maize yield, and this effect was amplified over time (in Figure 4a, the vertical gap between the two linear trend lines with CO_2_ or no CO_2_ gradually expanded), while the uncertainty range was smaller than that without CO_2_. Studies on rice yield changes are the most abundant. Rice yield change will be positive in the next few decades when CO_2_ is considered (that is, yield change (%) >0 with CO_2_), but the uncertainty interval was larger than that without CO_2_. Regarding wheat, there was a lack of consideration of CO_2_ factors (only two of the yield change results with CO_2_ were in the 2050s). However, overall, the wheat yield would not change much in the upcoming decades, which was a positive impact (yield change (%) >0) before the 2040s, and the positive impact decreased (the slope of the regression trend line was negative); after the 2050s, the impact was negative.

#### 3.3.2. Consensus Trend of Yield Change with Crops

For maize (Figure 4b), more than 50% of projections indicated yield increases in the 2030s and 2040s (yield change = 0–5%, MC), and more than 60% of all projections indicated yield decreases greater than 10% (yield change = −25 to −10% without CO_2_, HC; yield change = 0 to −25% with CO_2_, MC) in the 2050s. The magnitude of the yield impact increased with time, and approximately 89% and 74% of the projections indicated yield decreases to a large degree in the 2070s and 2080s, respectively. In addition, the yield changes still decrease with the impact of CO_2_ in the 2070s and 2080s, with yield changes of approximately 0 to −10% (95%, HC) in the 2070s and 0 to −5% (70%, HC) in the 2080s.

For rice (Figure 4d), before the 2050s many projections indicate yield decreases, but the range of decline was small. However, more predictions indicate that the yield change will be positive with CO_2_ factors. However, after the 2050s, more projections showed a larger decrease in the range for rice yield, and under CO_2_ factors, there is a higher consensus level of the yield change in the LC. There is an MC level of rice yield decreasing in the 2020s (60%) and 2030s (50%), and the higher consensus level shows that the yield would increase under CO_2_, which was approximately 10% to 25% (46%, MC). A yield change of −5 to 5% (MC) occurs in the 2040s and 2050s. After the 2060s, most projections show a high consensus level of yield decreases, with more than 85% of projections indicating a yield decrease greater than 10% in the 2090s.

For wheat (Figure 4e), the yield fluctuations in the upcoming decades were less volatile. For example, 76% and 72% of the projected yield changes were considered to vary from −5% to 5% in the 2020s and 2030s (HC), respectively, and more than 79% of the projections indicate yield decreases in the 2040s. The magnitude of the yield impact increases with time, and approximately 80% of the projections indicate yield decreases from −5 to −10% in the 2090s (HC).

Combined with the temporal trend and consensus analysis of each crop yield change, the following conclusions can be drawn: (i) The biggest impact of future climate change on crop yields in China occurs for maize, followed by rice and wheat. This result is reflected by the central tendency of yield change, which was −3.4% and −2.04% per 10 years for maize, −2.7% and −3.3% per 10 years for rice, and −1.4% per 10 years for wheat (there were no studies on wheat under the influence of CO_2_). (ii) Whether the CO_2_ factor was considered not only affected the maize and rice yield change rate, but also reduced the uncertainty range of the estimated results of the maize yield and increased the range of rice. (iii) The yield changes of maize decreases without CO_2_, and the yields decrease by 19% (high consensus) in the 2070s. (iv) The projections of rice yield declined with decade. In addition, the rice yields are negatively impacted without CO_2_ after the 2040s, and the yield decreases by approximately 10.4% (MC) in the 2060s. However, when considering the CO_2_ factor, there is a positive impact on the rice yield, similar to that in the 2050s, and the yield increases by approximately 10.86% (HC). (v) Wheat yield changes are relatively stable and decrease over time. Beginning with a reduction of 0.04% (without CO_2_; MC) in the 2030s, the wheat yield gradually declines, but the decline remains within 0–25%.

Through the above analysis, we can find that the consensus research can improve the reliability of the conclusion about crop yield change under future climate change to some extent. Without consensus analysis, it is difficult to clearly determine the quantitative conclusion of yield change from so many inconsistent conclusions. However, due to the differences in the negative changes of different crop yields, and the significant differences in regional environment and climate in the future, it is necessary for different regions to provide targeted measures and technologies to deal with future climate change. For example, for Northern China, which will face a water shortage in the future [20], it is necessary to improve the efficiency of crop irrigation as soon as possible and grow cultivars with higher water utilization efficiency [11]. Because of the wide distribution of rice, different adaptive techniques are needed in different regions, and studies have shown that growing cultivars with high heat requirements and adjusting the sowing date are two feasible approaches [28].

## 4. Discussion

In this paper, we comprehensively analyzed future crop yield changes in China based on many result samples of with the theme of climate change affecting China’s crop yields from the consensus of space and time. From results, there is a high consensus that yields will decrease sharply after 2050, with the decline trend after 2070 about five times that of around 2030. Hence, it is necessary to carry out scientific experiments and research on improving crop planting technology and cultivating new crop cultivars. It has been found that cultivating new cultivars, changing planting techniques and regional selective planting are the main viewpoints that are beneficial to the change of crop yield [7,16]. China is a vast country with complex terrain and climate, so the universal adaptation scheme cannot be applied to the whole research area [19]. For example, rice is planted in a wide range, including irrigation cultivation and rain-fed cultivation [15,17], while, the negative impact of climate change on rice is particularly significant, so it has been suggested that rice could be adapted to climate change by breeding rice cultivars with high temperature tolerance [26], extending the grout period and adjusting the sowing date [28]. Maize is mainly distributed in North China and Northeast China [38], however, studies have shown that northern China faces a high probability of water shortage due to the future climate change [20], so improving irrigation efficiency and switching to better water-use cultivars could mitigate the adverse effects of climate change on maize yields [11]. Moreover, winter wheat in Eastern China may have a shorter growing season due to the impact of warming, so delayed sowing is beneficial to the adaptability of wheat growth [19]. In this paper, we consider that the climatic factors affecting future yield mainly include rainfall, temperature and CO_2_. From the results, it can be found that the existing studies fully indicate that CO_2_ has a positive effect on the change of crop yield under appropriate conditions [16,22,28], but at higher CO_2_ concentration, the negative effect of warming will cancel out the fertilizer effect of CO_2_ [17], and different crops react differently to CO_2_. Therefore, it is necessary to further clarify the response of different crops to CO_2_ under different emission scenarios.

Negative change and regional differences in the future crop yield will affect the pattern of agricultural trade among regions in China, the government should make effective adjustments in the production, storage and transportation, balance the relationship between supply and demand among regions, and avoid a sharp rise in the price of agricultural products in areas where grain output has declined significantly [27], and it is necessary to actively construct and adjust commodity grain producing areas. In addition, the negative change and development of the future crop yield, will have a negative impact to food security and social welfare, and with trade links between agriculture and other economic sectors increasingly close, the other sectors are bound to be affected to varying degrees, further amplifying the negative impact of climate change on the agricultural sector [36,39,40]. Therefore, future research needs to combine climate change with socio-economic drivers to explore the interaction among climate change, crop yield and socio-economic development, which is of great significance for addressing climate change, ensuring future food security and national economic stability [35,36].

The data samples in this paper are from existing research results. Limited by these samples, we did not consider the influence of non-climate change factors, such as planting area, technology, adaptability, etc. There are few works in the literature that study the direct impact of extreme climatic conditions on crop yield, so we did not adopt the results of yield changes under extreme climatic conditions in this paper. As the frequency of extreme weather events increases in the future, studying the impact of extreme weather events on crop yields and food security risks is another topic we will continue to focus on. The consistent conclusion obtained by applying the likelihood scale and trend analysis method in this paper is reasonable and reliable, but how to expand the research method and improve the accuracy of the result uncertainty is the direction we need to learn about and improve.

## 5. Conclusions

Based on these articles on the main crop yield changes in China due to climate change, this paper evaluates the level of consensus on the reliability of the results in space and time. The high-consensus conclusions are as follows:

The crop yield change in China will be negative from the 2020s. Take 2012 as the baseline, the yield will decrease by 5% in the 2030s, and the decrease will be greater than 25% in the 2070s. The decline in the second half of this century will be greater than that in the first half.

Different crops respond differently to climate change. Maize yield will decrease more than 10% in the 2050s and approximately 19% in the 2070s. Rice yield will decrease faster in the second half, with its yield change decreasing from 5% to 25% after the 2060s. The fluctuations in wheat yield in upcoming decades will be less volatile, and the yield decrease will be approximately 10% in the 2090s.

CO_2_ factors have a positive impact on yield changes. The crop yield will decrease by 6.6% in the 2040s, but it will be increased by 15% with CO_2_. In the 2090s, the yield decrease will be 22.8%, but it will be 17.8% with CO_2_. In addition, the central tendency of maize and rice yield change will be −3.4% and −2.7% per 10 years, but they will change to +1.4% and −0.6% per 10 years, respectively, under the impact of CO_2_.

Furthermore, these studies are mostly concentrated in the NEC, CC and EC and need to be more inclusive of WC and SC. There are few studies of wheat yield change considering CO_2_ factors.

## Figures and Tables

**Table 1:** Statistical results of sample size under different conditions of different crops.

Crops	Without CO_2_	With CO_2_	All
Maize	149	66	215
Rice	155	88	243
Wheat	277	2	279
All	581	156	737

**Table 2:** Likelihood scale and consensus level.

Term	Virtually Certain	Very Likely	Likely	About as Likely as Not	Unlikely	Very Unlikely	Exceptionally Unlikely
Likelihood of the outcome (%)	99–100	90–99	66–90	33–66	0–33	1–10	0–1
Consensus level	Highest (HtC)		High (HC)	Medium (MC)	Low (LC)	Lowest (LtC)	

## Supplementary Materials

The following are available online at https://www.mdpi.com/1660-4601/17/24/9241/s1, Table S1: Information about SAs; File S1: The process of extracting and processing result samples from SAs. References [7,11,15,17,18,19,20,21,22,23,26,27,28,29,30,31,32,33,34,35,36] are cited in the supplementary materials.
