# Supplementary material for: What Is the Consensus from Multiple Conclusions of Future Crop Yield Changes Affected by Climate Change in China?
Source: Int J Environ Res Public Health. 2020 Dec 10;17(24):9241. doi: 10.3390/ijerph17249241 (PMC7763021; doi:10.3390/ijerph17249241)
Supplement: Supplementary file 1 [file ijerph-17-09241-s001.pdf]

Supplementary materials

The basic information of the 21 selected articles (SAs) in Table A1

Table S1. Information about SAs

NO.	Author	Title	Journal	Crop	Model
1	Liu et al. [1]	Reassessing Climate Change Impacts on Agriculture in China	Chinese Journal of Urban and Environmental Studies	Maize<br>Rice<br>Wheat	Statistical model
2	Wang et al. [2]	Effects of climate change on maize production, and potential adaptation measures: a case study in Jilin Province, China	Climate Research	Maize	CERES-Maize
3	Xiong et al. [3]	Future cereal production in China: The interaction of climate change, water availability and socio-economic scenarios	Global Environmental Change	Maize<br>Rice<br>Wheat	CERES
4	Yao et al. [4]	Assessing the impacts of climate change on rice yields in the main rice areas of China	Climatic Change	Rice	CERES-Rice
5	Jiang et al. [5]	Response of rice production to climate change based on self-adaptation in Fujian Province	Journal of Agricultural Science	Rice	CERES-Rice
6	Tao et al. [6]	Projected Crop Production under Regional Climate Change Using Scenario Data and Modeling: Sensitivity to Chosen Sowing Date and Cultivar	Sustainability	Wheat	WOFOST model
7	Song and Zhao [7]	Effects of Drought on Winter Wheat Yield in North China During 2012-2100	Acta Meteorologica Sinica	Wheat	WOFOST model
8	Kang et al. [8]	Predicting Climate Change Impacts on Maize Crop Productivity and Water Use Efficiency in the Loess Plateau	Irrigation and Drainage	Maize	SWAGMAN Destiny model

9	Wang et al. [9]	Responses of rice yield, irrigation water requirement and water use efficiency to climate change in China: Historical simulation and future projections	Agricultural Water Management	Rice	ORYZA2000
10	Xiong et al. [10]	Future cereal production in China: The interaction of climate change, water availability and socio-economic scenarios	Global Environmental Change	Maize<br>Rice<br>Wheat	CERES
11	Wu et al. [11]	CERES-Rice model-based simulations of climate change impacts on rice yields and efficacy of adaptive options in Northeast China	Crop & Pasture Science	Rice	CERES-Rice
12	Xin et al. [12]	The impacts of climate change on the People's Republic of China's grain Regional and crop perspective output:	China Agricultural Economic Review	Maize<br>Rice<br>Wheat	Statistical model
13	Lv et al. [13]	Climate change impacts on regional winter wheat production in main wheat production regions of China	Agricultural and Forest Meteorology	Wheat	Wheat Grow model
14	Zhao et al. [14]	Climatic potential productivity of winter wheat and summer maize in Huanghuaihai Plain in 2011-2050.	Chinese Journal of Applied Ecology	Maize<br>Wheat	AEZ
15	Tang et al. [15]	Estimating the potential yield and ETc of winter wheat across Huang- Huai-Hai Plain in the future with the modifed DSSAT model	scientific report	Wheat	DSSAT-CERES
16	Yang et al. [16]	Uncertainty of ensemble winter wheat yield simulation in North China based on CMIP5	Progress in Geography	Wheat	CERES-wheat
17	Yang et al. [17]	Impacts of Climate Change on Wheat Yield in China Simulated by CMIP5 Multi-Model Ensemble Projections	Scientia Agricultura Sinica	Wheat	CERES-wheat
18	Lv et al. [18]	Climate change impacts on regional rice production in China	Climatic Change	Rice	CERES-Rice
19	Zhang. et al.[19]	Using APSIM to explore wheat yield response to climate change in the North China Plain: The predicted adaptation of wheat cultivar types to vernalization.	Journal of Agricultural Science	wheat	CERES-Maize

20	Ye et al. [20]	Climate change impact on China food security in 2050	Agronomy for Sustainable Development	Maize<br>Rice<br>Wheat	CERES
21	Xiong et al. [21]	Modelling China's potential maize production at regional scale under climate change	Climatic Change	Maize	CERES-Maize

File S1: The process of extracting and processing result samples from SAs

There were some differences in SAs: (i) Differences in yield change results included different baselines, different quantitative expressions, and inconsistent predicted time periods. (ii) Different levels of study areas included national, regional, provincial, municipal and others. In response to these differences, we dealt with the data from SAs as follows.

(1) Baseline setting and assumption

The data obtained from SAs were expressed in two ways: absolute and relative change of crop yields compared with the baseline yield. For comparability, we needed to process a large amount of data. Under the assumption that the rate of yield change is constant, we converted the SA data to a new crop yield change (Y_{new}) with 2012 as the baseline. Meanwhile, the 2012 crop yield value was obtained from the National Bureau of Statistics of China (website: http://data.stats.gov.cn/easyquery.htm?cn=E0103). Then, the Y_{new} could be calculated by the formula below:

$$Y_{new} = \begin{cases} Y_{old} \times \dfrac{b_{2012}}{b_x} \\ (Y_{old\%} \times b_x + b_x) \times \dfrac{b_{2012}}{b_x} \end{cases} \quad \text{(B-1)}$$

where Y_{new} is the new yield change value with the baseline is 2012; Y_{old} and $Y_{old\%}$ represent the already given yield change value in SAs, and $Y_{old\%}$ indicates the percent change; and b_{2012} and b_x are the yield value in the baseline year, b_{2012} is the yield value in 2012, and b_{2012} is the yield value in the given x year in SAs.

(2) Unified the time periods in the future

The median time interval given by the articles was used; for example, if the data from one article were for 2040-2060, we considered the conclusion value to be for 2050. However, if the conclusion of the article was a yield change in a given year, the study directly used this time interval. Subsequently, we used an interdecadal period to set the time periods in the future; thus, the years 2020 and 2025 were in the 2020s, 2030 and 2035 were in the 2030s, etc. Ultimately, the time periods set in this study were 2020s=2020-2029, 2030s=2030-2039, …, 2090s=2090-2099.

(3) Definition of regions

This article uses 7 regions (shown in Fig. 1 in the main document) in China as research areas, regardless of the smaller spatial extent. Therefore, we applied the data of each province and city from the SAs directly to the region where they were located.

The prediction of crop yield change under future climate change in SAs was mostly based on the factors in different future greenhouse gas emissions scenarios (the Representative Concentration Pathways, RCPs) reported by the IPCC. However, this study did not involve the differentiation of these scenarios. We only analyzed and studied the results from SAs. Therefore, after the above data processing, we used the results of the crop yield changes at different times, different scenarios, and different research areas in SAs as sample data. For example, if an article predicted the yield changes for 2020 and 2040 for maize, rice, and wheat in regions A, B and C under the RCP4.5 and RCP8.5, then the paper was considered to provide 36 ($2(\text{scenarios})\times 3(\text{regions})\times 3(\text{crops})\times 2(\text{years})=36$) sample data sets. Then, we obtained 737 sets of yield change data from the 21 SAs (Table 2 in the main document)

Reference:

(1) Liu, J.; Liu, C.; Wen, Y. Reassessing Climate Change Impacts on Agriculture in China. *Chinese J. Urban Environ. Stud.* **2015**, *03* (02), 1550011. https://doi.org/10.1142/s2345748115500116.

(2) Wang, M.; Li, Y.; Ye, W.; Bornman, J. F.; Yan, X. Effects of Climate Change on Maize Production, and Potential Adaptation Measures: A Case Study in Jilin Province, China. *Clim. Res.* **2011**, *46* (3), 223–242. https://doi.org/10.3354/cr00986.

(3) Xiong, W.; Conway, D.; Lin, E.; Xu, Y.; Ju, H.; Jiang, J.; Holman, I.; Li, Y. Future Cereal Production in China: The Interaction of Climate Change, Water Availability and Socio-Economic Scenarios. *Glob. Environ. Chang.* **2009**, *19* (1), 34–44. https://doi.org/10.1016/j.gloenvcha.2008.10.006.

(4) Yao, F.; Xu, Y.; Lin, E.; Yokozawa, M.; Zhang, J. Assessing the Impacts of Climate Change on Rice Yields in the Main Rice Areas of China. *Clim. Change* **2007**, *80* (3–4), 395–409. https://doi.org/10.1007/s10584-006-9122-6.

(5) Jiang, M.; Shi, C. L.; Liu, Y.; Jin, Z. Q. Response of Rice Production to Climate Change Based on Self-Adaptation in Fujian Province. *J. Agric. Sci.* **2017**, *155* (5), 751–765. https://doi.org/10.1017/S0021859617000016.

(6) Tao, S.; Shen, S.; Li, Y.; Wang, Q.; Gao, P.; Mugume, I. Projected Crop Production under Regional Climate Change Using Scenario Data and Modeling: Sensitivity to Chosen Sowing Date and Cultivar. *Sustain.* **2016**, *8* (3), 1–23. https://doi.org/10.3390/su8030214.

(7) Song, Y.; Zhao, Y. Effects of Drought on Winter Wheat Yield in North China during 2012-2100. *Acta Meteorol. Sin.* **2012**, *26* (4), 516–528. https://doi.org/10.1007/s13351-012-0410-4.

(8) Kang, Y.; Ma, X.; Khan, S. Predicting Climate Change Impacts on Maize Crop Productivity and Water Use Efficiency in the Loess Plateau. *Irrig. Drain.* **2014**, *63* (3), 394–404. https://doi.org/10.1002/ird.1799.

(9) Wang, W.; Yu, Z.; Zhang, W.; Shao, Q.; Zhang, Y.; Luo, Y.; Jiao, X.; Xu, J. Responses of Rice Yield, Irrigation Water Requirement and Water Use Efficiency to Climate Change in China: Historical Simulation and Future Projections. *Agric. Water Manag.* **2014**, *146*, 249–261. https://doi.org/10.1016/j.agwat.2014.08.019.

(10) Xiong, W.; Conway, D.; Lin, E.; Holman, I. Potential Impacts of Climate Change and Climate Variability on China's Rice Yield and Production. *Clim. Res.* **2009**, *40* (1), 23–35. https://doi.org/10.3354/cr00802.

(11) Wu, W.; Fang, Q.; Ge, Q.; Zhou, M.; Lin, Y. CERES-Rice Model-Based Simulations of Climate Change Impacts on Rice Yields and Efficacy of Adaptive Options in Northeast China. *Crop Pasture Sci.* **2014**, *65* (12), 1267–1277. https://doi.org/10.1071/CP14009.

(12) Xin, X.; Lin, T.; Liu, X.; Wan, G.; Zhang, Y. The Impacts of Climate Change on the People's Republic of China's Grain Output: Regional and Crop Perspective. *China Agric. Econ. Rev.* **2013**, *5* (4), 434–458. https://doi.org/10.1108/CAER-03-2013-0049.

(13) Lv, Z.; Liu, X.; Cao, W.; Zhu, Y. Climate Change Impacts on Regional Winter Wheat Production in Main Wheat Production Regions of China. *Agric. For. Meteorol.* **2013**, *171–172*, 234–248. https://doi.org/10.1016/j.agrformet.2012.12.008.

(14) Zhao, J. F.; Guo, J. P.; Wu, D. R.; Fang, S. B.; E, Y. H. Climatic Potential Productivity of Winter Wheat and Summer Maize in Huanghuaihai Plain in 2011-2050. *Chinese J. Appl. Ecol.* **2011**, *22* (12), 3189–3195.

(15) Tang, X.; Song, N.; Chen, Z.; Wang, J.; He, J. Estimating the Potential Yield and ETc of Winter Wheat across Huang-Huai-Hai Plain in the Future with the Modified DSSAT Model. *Sci. Rep.* **2018**, *8* (1), 1–12. https://doi.org/10.1038/s41598-018-32980-4.

(16) Yang, X.; Tang, X.; Chen, B.; Tan, Y.; Tian, Z. Uncertainty of Ensemble Winter Wheat Yield Simulation in North China Based on CMIP5. *Prog. Geogr.* **2013**, *32* (4), 627–636. https://doi.org/10.11820/dlkxjz.2013.04.015.

(17) Yang, X.; Xu, T.; Baode, C.; Zhan, T.; Sijian, Z. Impacts of Climate Change on Peanut Yield in China Simulated by CMIP5 Multi-Model Ensemble Projections. *Sci. Agric. Sin.* **2014**, *47* (15), 3009–3024. https://doi.org/10.3864/j.

(18) Lv, Z.; Zhu, Y.; Liu, X.; Ye, H.; Tian, Y.; Li, F. Climate Change Impacts on Regional Rice Production in China. *Clim. Change* **2018**, *147* (3–4), 523–537. https://doi.org/10.1007/s10584-018-2151-0.

(19) Zhang, Y.; Feng, L. P.; Wang, J.; Wang, E. L.; Xu, Y. L. Using APSIM to Explore Wheat Yield Response to Climate Change in the North China Plain: The Predicted Adaptation of Wheat Cultivar Types to Vernalization. *J. Agric. Sci.* **2013**, *151* (6), 836–848. https://doi.org/10.1017/S0021859612000883.

(20) Ye, L.; Xiong, W.; Li, Z.; Yang, P.; Wu, W.; Yang, G.; Fu, Y.; Zou, J.; Chen, Z.; Van Ranst, E.; Tang, H. Climate Change Impact on China Food Security in 2050. *Agron. Sustain. Dev.* **2013**, *33* (2), 363–374. https://doi.org/10.1007/s13593-012-0102-0.

(21) Xiong, W.; Matthews, R.; Holman, I.; Lin, E.; Xu, Y. Modelling China’s Potential Maize Production at Regional Scale under Climate Change. *Clim. Change* **2007**, *85* (3–4), 433–451. https://doi.org/10.1007/s10584-007-9284-x.
